# Enhancing the Thermoelectric Performance of GeSb_4_Te_7_ Compounds via Alloying Se

**DOI:** 10.3390/ma16093368

**Published:** 2023-04-25

**Authors:** Siyu Wang, Tong Xing, Tian-Ran Wei, Jiawei Zhang, Pengfei Qiu, Jie Xiao, Dudi Ren, Xun Shi, Lidong Chen

**Affiliations:** 1 State Key Laboratory of High Performance Ceramics and Superfine Microstructure, Shanghai Institute of Ceramics, Chinese Academy of Sciences, Shanghai 200050, China; 2 School of Physical Science and Technology, ShanghaiTech University, Shanghai 201210, China; 3 State Key Laboratory of Metal Matrix Composites, School of Materials Science and Engineering, Shanghai Jiao Tong University, Shanghai 200240, China

**Keywords:** thermoelectric, GeSb_4_Te_7_, carrier concentration, alloying

## Abstract

Ge-Sb-Te compounds (GST), the well-known phase-change materials, are considered to be promising thermoelectric (TE) materials due to their decent thermoelectric performance. While Ge_2_Sb_2_Te_5_ and GeSb_2_Te_4_ have been extensively studied, the TE performance of GeSb_4_Te_7_ has not been well explored. Reducing the excessive carrier concentration is crucial to improving TE performance for GeSb_4_Te_7_. In this work, we synthesize a series of Se-alloyed GeSb_4_Te_7_ compounds and systematically investigate their structures and transport properties. Raman analysis reveals that Se alloying introduces a new vibrational mode of GeSe_2_, enhancing the interatomic interaction forces within the layers and leading to the reduction of carrier concentration. Additionally, Se alloying also increases the effective mass and thus improves the Seebeck coefficient of GeSb_4_Te_7_. The decrease in carrier concentration reduces the carrier thermal conductivity, depressing the total thermal conductivity. Finally, a maximum *zT* value of 0.77 and an average *zT* value of 0.48 (300–750 K) have been obtained in GeSb_4_Te_5.5_Se_1.5_. This work investigates the Raman vibration modes and the TE performance in Se-alloyed GeSb_4_Te_7_ sheddinglight on the performance optimization of other GST materials.

## 1. Introduction

Thermoelectric (TE) materials enable the direct interconversion of heat and electricity, which is extensively applied in the field of energy harvesting and device cooling [1,2,3,4]. The heat–electricity energy conversion efficiency is related to the dimensionless figure of merit *zT* (*zT* = *S*^2^*σ*/*κ*) of TE material, where *T* is the absolute temperature, *S* is the Seebeck coefficient, *σ* is the electrical conductivity, and *κ* is the thermal conductivity consisting of the lattice thermal conductivity (*κ_L_*) and carrier thermal conductivity (*κ_c_*) [5,6,7]. An ideal thermoelectric material should possess high *S* and large *σ*, combined with poor *κ* [8,9,10,11]. However, these electrical and thermal transport parameters are coupled with each other, so it is difficult to improve *zT* for optimizing them simultaneously. Therefore, a lot of research strategies for decoupling these parameters have been proposed to achieve high *zT* of TE materials [3,9,10,11,12].

As the pseudo-binary alloys of IV-VI and V_2_-VI_3_ tellurides, Ge-Sb-Te compounds (GSTs) have been widely used in rewritable storage techniques and are receiving more and more attention as TE materials. Metastable cubic and stable hexagonal phases are the two most common crystalline phases of GSTs. Due to their narrow band gap, excellent electrical conductivity, and low lattice thermal conductivity, stable hexagonal phase GSTs are the promising TE candidates, which exhibit anisotropic thermoelectric properties because of their layered structure [13]. In the previous works, Ge_2_Sb_2_Te_5_ and GeSb_2_Te_4_ have been widely studied and optimized to achieve excellent TE performance. By introducing resonance energy levels through In-doping, Hu et al. increased the effective mass of Ge_2_Sb_2_Te_5_ and obtained a maximum *zT* value of 0.78 at 700 K [14]. Moreover, the electrical properties of Ge_2_Sb_2_Te_5_ can also be successfully improved by modulation at the anion site [15,16]. For the GeSb_2_Te_4_ single crystal, In-doping also introduces an impurity band and results in the locally distorted density of states, which contributes to the enhanced Seebeck coefficient and improved power factor [17,18]. In the GST materials family, GeSb_4_Te_7_ gains less attention than Ge_2_Sb_2_Te_5_ and GeSb_2_Te_4_ in view of its poor intrinsic TE performance. GeSb_4_Te_7_ is a strongly degenerate p-type semiconductor and possesses an excessively high hole carrier concentration, limiting the improvement of TE performance. Our previous study focused on GeSb_4_Te_7_ and successfully decreased the carrier concentration via alloying the n-type homologous counterpart GeBi_4_Te_7_ [19]. However, the carrier concentration of GeSb_4_Te_7_ has not been adjusted to the optimal range, and its TE performance can be further improved.

In this study, we synthesized polycrystalline GeSb_4_Te_7−*x*_Se*_x_* through melting and SPS sintering and investigated the Raman vibration modes using both experimental and calculated Raman data. Our findings provide new insights into the vibrational modes of GeSb_4_Te_7_, including the discovery of a new vibrational mode introduced by Se alloying. This leads to the reduced carrier concentration and improved TE performance of GeSb_4_Te_7_. A peak *zT* of 0.77 at 750 K and an average *zT* of 0.48 between 300 and 750 K have been realized in GeSb_4_Te_5.5_Se_1.5_, which is 54% higher than that of the pristine GeSb_4_Te_7_. This study shows that the modified vibrational modes may provide a powerful strategy to optimize TE performance, making GST compounds to be promising TE materials.

## 2. Experimental Methods

### 2.1. Material Synthesis

GeSb_4_Te_7−*x*_Se*_x_* (*x* = 0, 0.1, 0.2, 0.3, 0.5, 0.8, 1.5, 2.0, and 2.5) samples were synthesized by the melting–annealing method. Raw materials with high purity, including Ge Shots, Sb Shots, Te Shots, and Se Shots (99.999%, Alfa Aesar), were weighed as designed chemical ratios and sealed in the vacuum quartz tubes. The tubes were heated to 1273 K and kept for 12 h. Next, they were quenched in ice water and annealed at 773 K for 3 days. Finally, the obtained ingots were ground into fine powders and consolidated using spark plasma sintering (SPS, Dr Sinter SPS-2040, Fuji Electronic Industrial Co., Ltd, Saitama, Japan) at 673–723 K for 5 min under a uniaxial pressure of 50 MPa. Pellets with a diameter of 10 mm were obtained, and their densities were higher than 98% of the theoretical density.

### 2.2. Calculation

The calculations were performed employing the projector augmented wave (PAW) method [20] combined with the generalized gradient approximation (GGA) [21] of the modified Perdew–Burke–Ernzerhof form for solids (PBEsol) [22] and the Vienna Ab initio Simulation Package (VASP) [23]. The crystal structure of GeSb_4_Te_7_ was obtained from the ICSD database (No. 42875). The space group is $P\overset{-}{3}m1$ and the lattice parameters are a = 4.21000(2) (Å) and c = 23.65000(80) (Å). The crystal structure was fully optimized with a 12 × 12 × 2 Monkhorst–Pack k mesh and a force convergence criterion of 1 meV Å^−1^. The phonon vibrational frequencies at the Γ point were calculated in supercells with 300 atoms (5 × 5 × 1 primitive cells) using the finite displacement method [24], which was implemented by combing VASP and Phonopy [25]. The atomic force calculations were conducted with a 2 × 2 × 2 Γ-centered *k*-mesh. All calculations were achieved with a plane-wave cutoff energy of 500 eV. A convergence criterion of 1 × 10^−6^ eV was adopted for the electronic loop.

### 2.3. Material Characterization

X-ray diffraction with Cu Kα radiation (XRD, D/max 2550 V, Rigaku, Tokyo, Japan) was performed to determine the crystal structures. Scanning electron microscopy (SEM, ZEISS supra55, Munich, Germany) equipped with EDS was employed to characterize the chemical components. The Raman spectrum of the GeSb_4_Te_7−*x*_Se*_x_* powder samples were recorded on an XploRA ONE-532 (Horiba, Kyoto, Japan). ZEM-3 (Ulac-Riko, Yokohama, Japan) was performed to measure the electrical conductivity (*σ*) and Seebeck coefficient (*S*) from 300 K to 750 K [12]. The thermal conductivity (κ) was obtained from the relation $\kappa ={\lambda \rho C}_{p}$ [9], where the thermal diffusion coefficient (*λ*) was obtained using the laser pulse analyzer (LFA457, Netzsch, Selb, Germany); the density (ρ) was measured by the Archimedes drainage method; the heat capacity *C*_p_ was calculated by the Dulong–Petit law. The Hall coefficient $R_{H}$ was obtained by the Physical Property Measure System (PPMS, Quantum Design, San Diego, CA, USA), using an AC magnetic field Hall test in the range −5 T–5 T. The carrier concentration was obtained according to $p_{H}=1/eR_{H}$, and the mobility was calculated based on $\mu_{H}=\sigma R_{H}$. The absorption spectra were obtained by measuring the powder samples using UV-Vis spectroscopy (Shimadzu Spectroscope, UV-3101PC, Kyoto, Japan) at room temperature [8]. The optical band gaps of the samples were calculated from the relationship between the optical band gap and the absorption coefficient ${\left(\alpha hv\right)}^{\frac{1}{n}}=B(hv-E_{g})$ [26].

## 3. Results and Discussion

### 3.1. Phase and Crystal Structures

**Figure 1:**
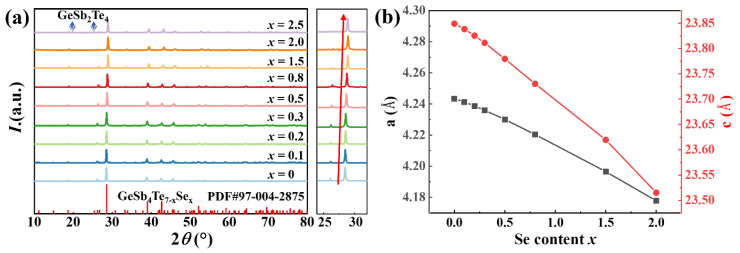
(**a**) Room temperature X-ray diffraction pattern of powder samples and (**b**) the lattice constants obtained by refinement as a function of Se content for GeSb_4_Te_7−*x*_Se*_x_* (*x* = 0, 0.1, 0.2, 0.3, 0.5, 0.8, 1.5, and 2.0).

The X-ray diffraction (XRD) patterns of GeSb_4_Te_7−*x*_Se*_x_* (*x* = 0, 0.1, 0.2, 0.3, 0.5, 0.8, 1.5, 2.0, and 2.5) were obtained at room temperature and are presented in Figure 1a. The diffraction peaks are well identified as belonging to the hexagonal structure ($P\bar{3}m1$) of GeSb_4_Te_7_. When the Se content is less than 2.0, no obvious secondary phase is observed within the detection limits. However, impurity peaks appear when *x* = 2.5, indexed to GeSb_2_Te_4_. With the increase of Se content, the diffraction peaks gradually shift towards the high angle, attributed to the small atomic radius of Se in comparison with Te. The lattice constants of the GeSb_4_Te_7−*x*_Se*_x_* samples were obtained by refining the XRD data, as shown in Figure 1b. The a- and c-axis lattice constants both show decreasing trends with increasing Se content, which is consistent with the XRD results. Furthermore, SEM and EDS analyses of GeSb_4_Te_5_Se_2_ are shown in Figure S1, confirming that all the elements are homogeneously distributed without obvious secondary phases or enriched phases. These results indicate that Se atoms enter the lattice of GeSb_4_Te_7_ and substitute the Te atoms.

### 3.2. Raman Measurement and Analysis

**Figure 2:**
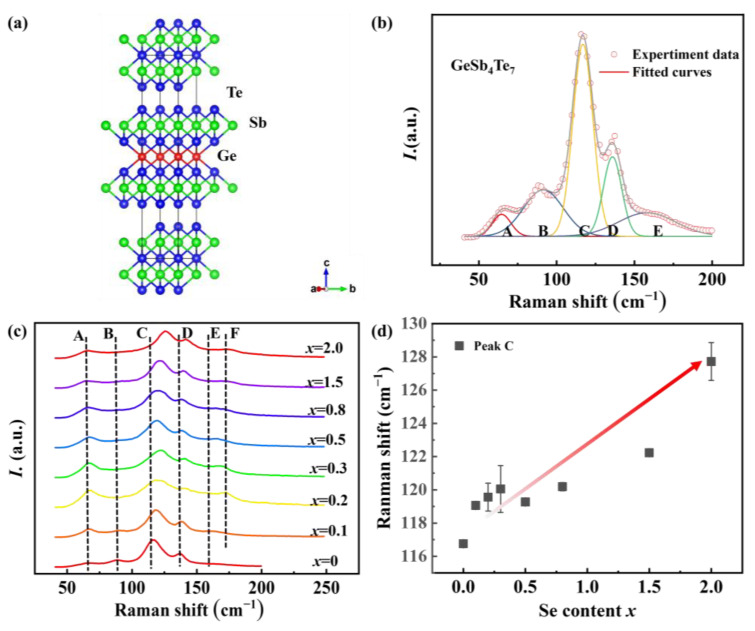
(**a**) The crystal structure of GeSb_4_Te_7_. (**b**) The experimental Raman spectra of GeSb_4_Te_7_ and the fitting curve using Gaussian model. (**c**) The experimental Raman spectra of GeSb_4_Te_7−*x*_Se*_x_* (*x* = 0, 0.1, 0.2, 0.3, 0.5, 0.8, 1.5, and 2.0). (**d**) The Raman shift of C peak as a function of Se content.

Raman scattering spectroscopy is an effective and highly sensitive tool for characterizing the crystal structure of materials since photons directly couple to the lattice vibrations that reflect the local crystal symmetry. To investigate the effect of Se on the original bonding environment, Raman spectroscopy analysis was conducted to identify the frequency of bonding vibrational modes among Ge, Sb, Te, and Se atoms. GeSb_4_Te_7_ possesses a hexagonal phase structure (D^3^_3d_ symmetry) with 10 Raman vibrational modes at the Γ point of the Brillouin zone [27]:

(1) $$\Gamma_{Raman}=5A_{1g}+5E_{g},$$

where the A_1g_ and E_g_ modes correspond to the intralayer and interlayer vibrations along the c-axis and are related to the stretching vibration and the birefringent vibration of atoms in the molecule, respectively (Figures S2 and S3). To study the crystal structure and vibrational modes of GeSb_4_Te_7_, we adopted a 5 × 5 × 1 supercell and calculated the theoretical frequencies of Raman vibrational modes employing the density functional theory (DFT), as shown in Table S1. Different from Ge_2_Sb_2_Te_5_, GeSb_4_Te_7_ consists of five-layer Sb_2_Te_3_ and seven-layer GeSb_2_Te_4_ along the c-axis (Figure 2a), and thus its Raman vibrational modes can be considered a superposition of several module vibrational modes [28]. The detailed theoretical analysis of Raman vibration modes is described in Supplementary Material.

Moreover, we fitted the experimental Raman spectrum of GeSb_4_Te_7_ using the Gaussian model (Figure 2b), and the detailed values of the fitted parameters are shown in Table S2. The A, B, C, and E peaks of the Raman spectrum for GeSb_4_Te_7_ correspond to the calculated vibrational modes A_1g_(2), E_g_(3), A_1g_(3), and A_1g_(4). The absence of the D peak in the calculated results may be attributed to the anisotropy of GeSb_4_Te_7_, which is also observed in the experimental Raman vibrational frequency of MnBi_4_Te_7_ with a similar structure [28]. The experimental Raman spectra of GeSb_4_Te_7−*x*_Se*_x_* are presented in Figure 2c. It is indicated that Se-alloying introduces a new Raman vibrational mode with a frequency of approximately ~162 cm^−1^ (Peak F), which corresponds to the GeSe_2_ [29]. With the increase of Se, the C peak generally shifts to the right direction, as plotted in Figure 2d, indicating that Se-alloying enhances the vibrational modes of A_1g_. This suggests that the substitution of Te by Se enhances the interatomic interactions within the layer and the bond strength between Ge and Te/Se, which affects the intrinsic Ge vacancy and decreases the hole carrier concentration. A similar phenomenon was observed in S-doped in Cu_2_Se [30]. It is noteworthy that the shape of Raman peaks is not changed, implying that the crystal structure and symmetry of GeSb_4_Te_7_ are scarcely altered via the introduction of Se.

### 3.3. Thermoelectric Properties

**Figure 3:**
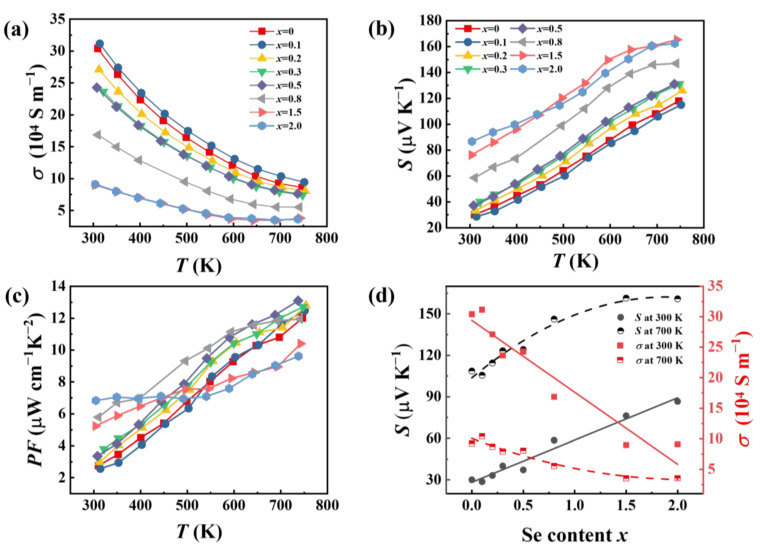
Temperature dependence of (**a**) electrical conductivity *σ*, (**b**) Seebeck coefficient *S*, and (**c**) power factor *PF* for GeSb_4_Te_7−*x*_Se*_x_* samples (*x* = 0, 0.1, 0.2, 0.3, 0.5, 0.8, 1.5, and 2.0). (**d**) *σ* and *S* at 300 K and 700 K as a function of the Se-alloying content.

The electrical conductivity (*σ*), Seebeck coefficient (*S*), and power factor (*PF*) of the GeSb_4_Te_7−*x*_Se*_x_* samples (*x* = 0, 0.1, 0.2, 0.3, 0.5, 0.8, 1.5, and 2.0) with the temperature are presented in Figure 3. All samples exhibit positive and metallic conducting behavior. The *σ* decreases continuously over the entire measured temperature range with the Se content increases. The *σ* for GeSb_4_Te_5.5_Se_1.5_ is 8.9 × 10^4^ S m^−1^ at 750 K, which is just about 30% of that for pristine GeSb_4_Te_7_. Moreover, Se alloying significantly enhances the Seebeck coefficient of GeSb_4_Te_7_. The *S* for GeSb_4_Te_5.5_Se_1.5_ is around 165 μV K^−1^ at 750 K, which is about 140% higher than that of pristine GeSb_4_Te_7_. Figure 3d illustrates the *S* and *σ* of GeSb_4_Te_7−*x*_Se*_x_* at 300 K and 700 K as a function of the Se-alloying content. However, the enhancement in *S* cannot compensate for the reduction in *σ*, resulting in a continuous decrease of the *PF* with increasing Se-alloying content at a higher temperature range. As shown in Figure 3c, the maximum *PF* for GeSb_4_Te_5_Se_2_ is 6.8 μW cm^−1^ K^−1^ at 300 K, about twice that of GeSb_4_Te_7_.

**Figure 4:**
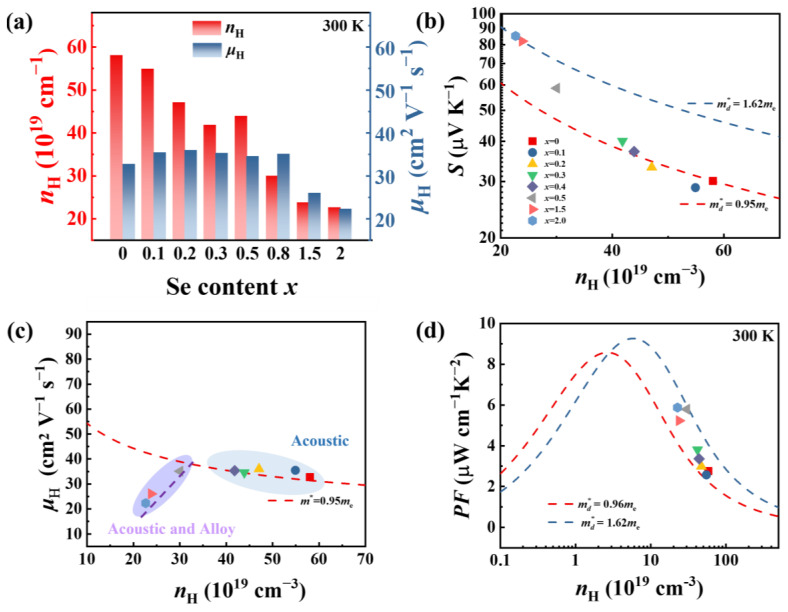
(**a**) The carrier concentration (*n*_H_) and mobility (*μ*_H_) at room temperature as a function of the Se alloying content. (**b**) *S*, (**c**) *μ*_H_, and (**d**) *PF* versus carrier concentration *n* for GeSb_4_Te_7−*x*_Se*_x_* at 300 K. The red and blue dashed lines represent the predicted values based on the SPB model with different effective masses.

The carrier concentration (*n*_H_) and mobility (*μ*_H_) were measured to further understand the electrical transport properties of GeSb_4_Te_7−*x*_Se*_x_* samples. Upon the introduction of Se into GeSb_4_Te_7_, the carrier concentration (*n*_H_) at 300 K reduces from 5.8 × 10^20^ cm^−3^ to 2.4 × 10^20^ cm^−3^, as shown in Figure 4a. This reduction of *n*_H_ is attributed to the enhanced interatomic forces within the layer via the introduced vibrational modes of GeSe_2_ upon Se alloying. The suppressed *n*_H_ is responsible for the decreased electrical conductivity and the enhanced Seebeck coefficient (Figure 3d). However, the carrier mobility (*μ*_H_) hardly changes with increasing Se (*x* ≤ 0.8). When the Se content is more than 0.8, the *μ*_H_ shows a significant decrease, and it is only 26.0 cm^−2^V^−1^s^−1^ for *x* = 1.5, which is approximately 68% of that for the pristine GeSb_4_Te_7_.

The single parabolic band (SPB) model was employed to analyze the modification of electrical transport properties for GeSb_4_Te_7−*x*_Se*_x_*. The transport parameters can be expressed as follows [31,32,33]:

(2) $$S=\frac{k_{B}}{e}(\frac{2F_{1}}{F_{0}}-\eta ),$$

(3) $$n_{H}=\frac{8\pi {\left(2m_{d}^{*}T\right)}^{\frac{3}{2}}}{3h^{3}}\frac{2F_{0}^{2}}{F_{-1/2}},$$

(4) $$\mu_{H}=\mu_{0}\frac{F_{-1/2}}{2F_{0}},$$

(5) $$PF=S^{2}n_{H}\mu_{H}e,$$

where *η* is the reduced Fermi level, $F_{i}(\eta )$ is the Fermi integral expressed by $F_{i}(\eta )=\int_{0}^{\infty }\frac{x^{2}dx}{1+\exp(x-\eta )}$, m* is the effective mass, *e* is the elementary charge, $k_{B}$ is the Boltzmann constant, and *h* is the Planck constant. Figure 4b plots the Pisarenko relationship (*S* vs. *n*) for GeSb_4_Te_7−*x*_Se*_x_* (*x* = 0, 0.1, 0.2, 0.3, 0.5, 0.8, 1.5, and 2.0). The *S* decreases with the increasing *n*_H_ at 300 K. Based on Equations (2) and (3), the Pisarenko curves with different m* are also plotted in Figure 4b. For the pristine GeSb_4_Te_7_, the m* is 0.95 *m_e_* at 300 K (*m_e_* is the free electron mass). The experimental data of all Se-alloyed samples are between two calculated dashed lines with m*  =  0.92 *m_e_* and 1.62 *m_e_*, which are derived under the single parabolic band (SPB) model [31,32,34]. This indicates that Se-alloying increases the DOS near the Fermi energy level of GeSb_4_Te_7_. A similar phenomenon was also observed in Se-alloyed Ge_2_Sb_2_Te_5_ [15]. Furthermore, the band gaps of the GeSb_4_Te_7−*x*_Se*_x_* samples were measured by the optical diffuse reflectance spectrum and listed in Table S4, indicating that Se-alloying affects the band structure and slightly decreases the band gap of GeSb_4_Te_7_.

We also investigated the relationship between *n* and *μ*_H_ for GeSb_4_Te_7−*x*_Se*_x_* at 300 K, which is illustrated in Figure 4c. The red dashed line is the theoretical curve considering only acoustic phonon scattering based on the SPB model for m* = 0.95 *m*_e_, and the purple dashed line is the theoretical curve considering acoustic phonon and alloying scattering. For samples with *x* less than 0.8, acoustic phonon scattering is the dominant scattering mechanism for carriers, and the mobility is calculated based on acoustic phonon scattering following the red dotted line. However, when *x* ≥ 0.8, alloying scattering is not negligible anymore, alloying scattering combined with acoustic phonon scattering is considered. When the carriers are dominantly scattered by both acoustic phonons and alloy scattering, *μ*_0_ is provided by [30,35]:

(6) $$\frac{1}{\mu_{0}}=\frac{1}{\mu_{ac,0}}+\frac{1}{\mu_{al,0}},$$

(7) $$\mu_{ac,0}=\frac{\pi e\hbar^{4}dv_{l}^{2}}{\sqrt{2}\Xi^{2}m_{b}^{*5/2}{\left(k_{B}T\right)}^{3/2}},$$

(8) $$\mu_{al,0}=\frac{8e\hbar^{4}N_{0}}{3\sqrt{2}\pi x(1-x)m_{b}^{*5/2}{\left(k_{B}T\right)}^{1/2}U^{2}},$$

where *μ*_ac,0_ and *μ*_al,0_ are the mobilities for acoustic phonon scattering and alloy scattering, respectively. The mobilities of the samples with *x* ≥ 0.8 obviously deviate from the red dashed line. The decrease in *μ*_H_ with increasing Se-alloying content in Figure 4a is attributed to the additional alloy scattering. The *PF* and *n*_H_ data for GeSb_4_Te_7−*x*_Se*_x_* at 300 K are plotted in Figure 4d. The experimental data fall between the theoretical Pisarenko curves with m* = 0.95 *m*_e_ and m* = 1.62 *m*_e_. Se alloying in GeSb_4_Te_7_ decreases the carrier concentration and achieves higher *PF.*

**Figure 5:**
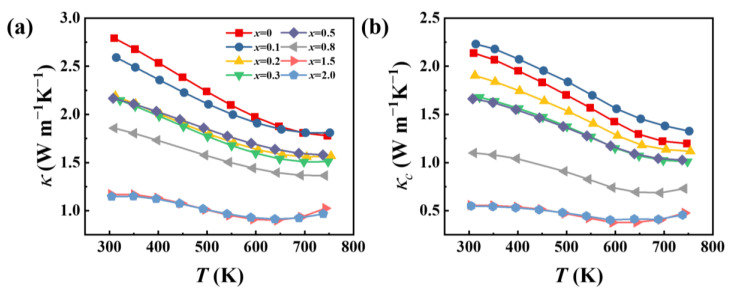
Temperature dependence of (**a**) thermal conductivity *κ* and (**b**) carrier thermal conductivity *κ*_c_ for the GeSb_4_Te_7−*x*_Se*_x_* (*x* = 0, 0.1, 0.2, 0.3, 0.5, 0.8, 1.5, and 2.0).

In addition to the electrical transport properties, the thermal transport properties of GeSb_4_Te_7_ are also strongly affected by alloying Se. The thermal conductivities (*κ*) of all GeSb_4_Te_7−*x*_Se*_x_* samples are presented in Figure 5a. With the increasing Se alloying content, the *κ* gradually decreases. The *κ* of GeSb_4_Te_5.5_Se_1.5_ is approximately 1.2 W m^−1^K^−1^ at 300 K, roughly 43% of GeSb_4_Te_7_. Such a reduction is mainly attributed to the suppression of the carrier thermal conductivity (*κ*_c_). The *κ*_c_ is calculated based on the Wiedeman–Franz law and presented in Figure 5b (*κ*_c_ = *L*_0_*Tσ*, where *L*_0_ is calculated based on the single parabolic band model) [36,37,38]. The *κ*_c_ decreases with increasing Se alloy content over the temperature range. The lattice thermal conductivities *κ_L_* (*κ_L_* = *κ* − *κ*_c_) of GeSb_4_Te_7−*x*_Se*_x_* samples are also calculated and presented in Figure S4. The *κ_L_* decreases first and then rises with increasing Se content. A similar phenomenon is observed in the Se-alloyed Ge_2_Sb_2_Te_5_ [15]. The minimum *κ_L_*, about ~0.26 Wm^−1^K^−1^ at 400 K, is obtained in GeSb_4_Te_6.8_Se_0.2_. The reduction of *κ_L_* is primarily caused by the differences in atomic radii and mass between Te and Se atoms, which introduces a strong strain field and mass fluctuations.

**Figure 6:**
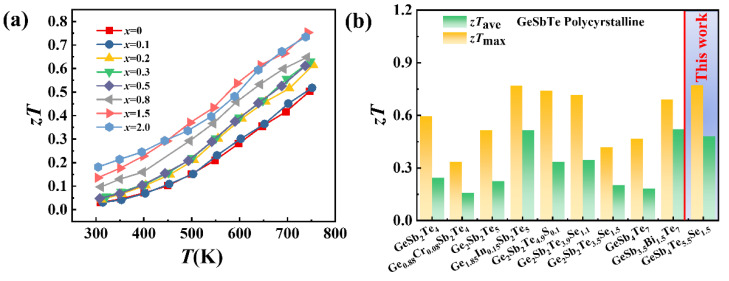
(**a**) Temperature dependence of TE figure of merit *zT* for the GeSb_4_Te_7−*x*_Se*_x_* (*x* = 0, 0.1, 0.2, 0.3, 0.5, 0.8, 1.5, and 2.0). (**b**) Comparison of the maximum and average *zT* values at 300–750 K for several GST compounds and GeSb_4_Te_5.5_Se_1.5_ in this work [13,14,15,17,19,39].

The TE figure of merit *zT* for the GeSb_4_Te_7−*x*_Se*_x_* samples is presented in Figure 6a. The *zT* is obviously enhanced upon alloying Se over the entire measured temperature range, attributed to the improved Seebeck coefficient and suppressed thermal conductivity. When *x* = 1.5, a maximum *zT* value of 0.77 is achieved at 750 K, about 50% higher than that of pristine GeSb_4_Te_7_. Moreover, the average *zT* value within the temperature range 300–750 K for GeSb_4_Te_5.5_Se_1.5_ is 0.48. These values are among the highest values reported in GST compounds, as shown in Figure 6b [13,14,15,17,19,39].

## 4. Conclusions

In summary, a series of polycrystalline GeSb_4_Te_7−*x*_Se*_x_* compounds were synthesized and investigated. Alloying Se suppresses the carrier concentration resulting from the enhancing interatomic interaction forces within the layers via the introduced new Raman vibrational modes. The Seebeck coefficient is improved because of the reduced carrier concentration and enhanced effective mass by Se alloying. In addition, the thermal conductivity is obviously decreased by the simultaneous reduction of *κ_L_* and *κ_c_*. Eventually, GeSb_4_Te_5.5_Se_1.5_ shows a peak *zT* value of 0.77 at 750 K and an average *zT* value of 0.48 within the temperature range of 300–750 K. This study deepens the understanding of GeSb_4_Te_7_ and provides a new approach for optimizing the TE performance of GST compounds via introducing new vibrational modes.

## Data Availability

Data is contained within the article or supplementary material.

## Supplementary Materials

The following supporting information can be downloaded at: https://www.mdpi.com/article/10.3390/ma16093368/s1. Figure S1: (a) Backscatter electron (BSE) image, (b) secondary electron (SE2) image of the fractured surface, and energy dispersive spectroscopy (EDS) mappings for (c) Ge, (d) Sb, (e) Te, (f) Se of GeSb_4_Te_5_Se_2_; Figure S2: Sketch of the displacement patterns of Raman-active E_g_ phonons at the Γ-point for GeSb_4_Te_7_. Displacements along the c axis are involved; Figure S3: Sketch of the displacement patterns of Raman-active A_1g_ phonons at the Γ-point for GeSb_4_Te_7_. Displacements along the c axis are involved; Figure S4: Temperature dependence of lattice thermal conductivity *κ*_L_ for GeSb_4_Te_7-*x*_Se*_x_* samples; Figure S5: (a) Mass change and (b) the surface of GeSb_4_Te_5_Se_2_ samples before and after annealing at 750 K; Table S1: Theoretically calculated and measured frequency (cm^−1^) of g-mode (Raman-active) phonons of the Γ-point phonons for optimized geometry GeSb_4_Te_7_; Table S2: Peak identity of Raman spectra of GeSb_4_Te_7_; Table S3: C-peak Raman shifts and linewidths of GeSb_4_Te_7-*x*_Se*_x_* with different Se contents; Table S4: Direct band gap of hexagonal GeSb_4_Te_7-*x*_Se*_x_* compounds. References [27,28,40,41] are cited in the supplementary materials.
