# Supplementary material for: Enhancing the Thermoelectric Performance of GeSb4Te7 Compounds via Alloying Se
Source: Materials (Basel). 2023 Apr 25;16(9):3368. doi: 10.3390/ma16093368 (PMC10180192; doi:10.3390/ma16093368)
Supplement: Supplementary file 1 [file materials-16-03368-s001.zip › materials-2322235-supplementary.pdf]

Supplementary Material

**Calculated Raman frequency**

In contrast to the of $Ge_2Sb_2Te_5$ [40], $GeSb_4Te_7$ consists of 12 layers, including a 5-layer $Sb_2Te_3$ module and a 7-layer $GeSb_2Te_4$ module along the c-axis, and thus its Raman vibrational modes can be considered as a superposition of several module vibrational modes. Comparing the calculated Raman vibrational modes of $Sb_2Te_3$ [27], $Ge_2Sb_2Te_5$ [40], $MnBi_2Te_4$ [28], and $GeSb_4Te_7$, we found that the frequencies and vibrational modes of the $E_g(2)$ (48.4 $cm^{-1}$), $A_{1g}(2)$ (66.9 $cm^{-1}$), $E_g(4)$ (109.5 $cm^{-1}$), and $A_{1g}(5)$ (167.6 $cm^{-1}$) modes of $GeSb_4Te_7$ correspond to those of the $E_g(1)$, $A_{1g}(1)$, Eg(2), and $A_{1g}(2)$ modes of $Sb_2Te_3$. The frequencies of the $E_g(1)$ (35.2 $cm^{-1}$), $A_{1g}(1)$ (47.9 $cm^{-1}$), $E_g(3)$ (103.1 $cm^{-1}$), and $A_{1g}(3)$ (113.3 $cm^{-1}$) modes of $GeSb_4Te_7$ match those of the $E_g(1)$, A1g(1), $E_g(3)$, $A_{1g}(2)$, and $A_{1g}(3)$ modes of $MnBi_2Te_4$, while the $E_g(5)$ (117.2 $cm^{-1}$) and $A_{1g}(5)$ (170.4 $cm^{-1}$) modes of $GeSb_4Te_7$ are similar to the $E_g(4)$ and $A_{1g}(4)$ modes of $Ge_2Sb_2Te_5$.

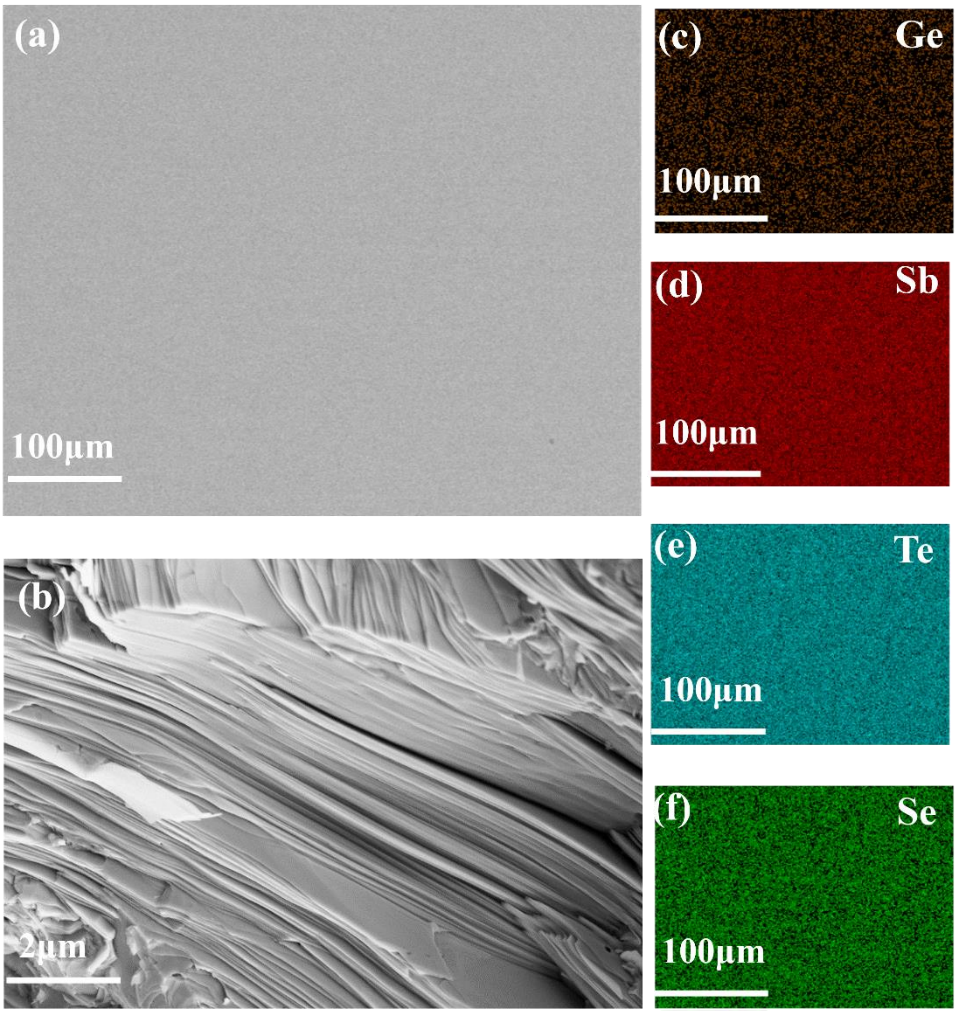


**Figure S1** (a) Backscatter electron (BSE) image, (b) secondary electron (SE2) image of the fractured surface, and energy dispersive spectroscopy (EDS) mappings for (c) Ge, (d) Sb, (e) Te, (f) Se of $GeSb_4Te_5Se_2$ sample.

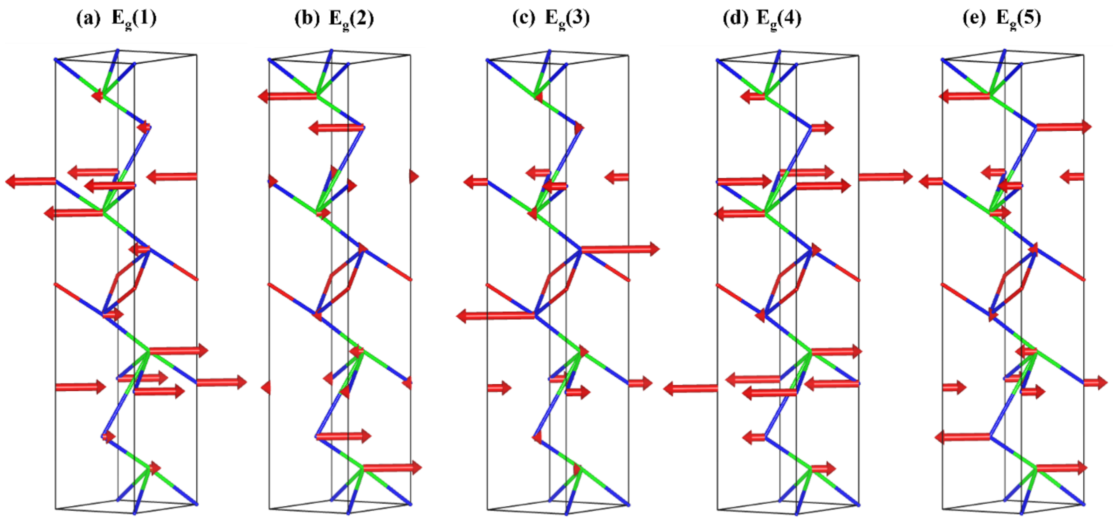


**Figure S2** Sketch of the displacement patterns of Raman-active $E_g$ phonons at the Γ-point for $GeSb_4Te_7$. Displacements along the c axis are involved.

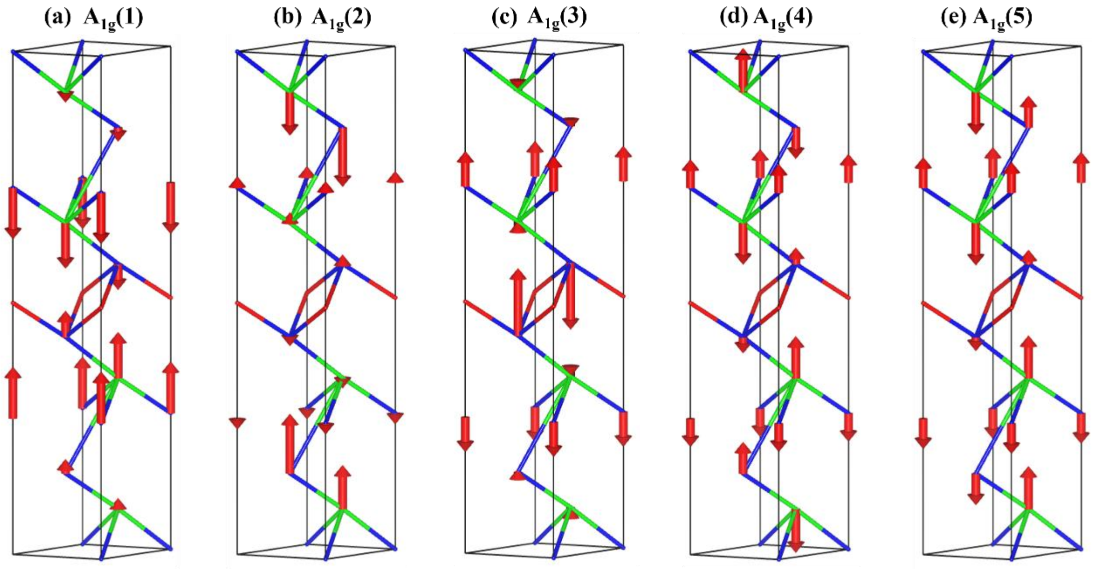


**Figure S3** Sketch of the displacement patterns of Raman-active $A1_g$ phonons at the Γ-point for $GeSb_4Te_7$. Displacements along the c axis are involved.

**Tabel S1** Theoretically calculated and measured frequency ($cm^{-1}$) of g-mode (Raman-active) phonons of the ***Γ***-point phonons for optimized geometry $GeSb_4Te_7$.

| Modes | Calculated ($cm^{-1}$) | Peak Identity | Measured ($cm^{-1}$) |
|---|---|---|---|
| **$E_g(1)$** | 35.2 | - | - |
| **$A_{1g}(1)$** | 47.9 | - | - |
| **$E_g(2)$** | 48.4 | - | - |
| **$A_{1g}(2)$** | 66.9 | A | 67.6 |
| **$E_g(3)$** | 103.1 | B | 93.6 |
| **$E_g(4)$** | 109.5 | - | - |
| **$A_{1g}(3)$** | 113.3 | C | 118.3 |
| **$E_g(5)$** | 117.2 | -- | - |
| **-** | - | D | 137.9 |
| **$A_{1g}(4)$** | 167.6 | E | 162.9 |
| **$A_{1g}(5)$** | 170.4 | - | - |

**Tabel S2** Peak identity of Raman spectra of $GeSb_4Te_7$.

| Peak Identity | ω($cm^{-1}$) | | Intensity | | FWHM($cm^{-1}$) | |
|---|---|---|---|---|---|---|
| | value | Standard Error | value | Standard Error | value | Standard Error |
| **Peak A** | 67.65 | 0.54 | 3.64 | 0.37 | 14.20 | 1.20 |
| **Peak B** | 93.58 | 1.02 | 5.64 | 0.79 | 24.71 | 3.99 |
| **Peak C** | 118.27 | 0.25 | 15.50 | 0.96 | 14.87 | 0.74 |
| **Peak D** | 137.90 | 0.47 | 8.93 | 0.80 | 17.34 | 1.45 |
| **Peak F** | 162.23 | 0.83 | 4.10 | 0.42 | 19.04 | 1.97 |

**Tabel S3** C-peak Raman shifts and linewidths of $GeSb_4Te_{7-x}Se_x$ with different Se contents.

| Se content x | ω(cm$^{-1}$) | | FWHM(cm$^{-1}$) | |
|---|---|---|---|---|
| | value | Standard Error | value | Standard Error |
| **0** | 117.09 | 0.13 | 14.85 | 0.74 |
| **0.1** | 118.26 | 0.24 | 15.39 | 0.48 |
| **0.2** | 119.30 | 1.17 | 18.42 | 4.25 |
| **0.3** | 120.42 | 0.63 | 17.45 | 2.76 |
| **0.5** | 119.18 | 0.29 | 17.00 | 1.08 |
| **0.8** | 118.07 | 1.30 | 14.81 | 2.45 |
| **1.5** | 121.29 | 0.29 | 16.72 | 0.92 |
| **2.0** | 126.04 | 0.38 | 17.75 | 0.99 |

**Table S4** Direct band gap of hexagonal $GeSb_4Te_{7-x}Se_x$ compounds.

| | $GeSb_4Te_{7-x}Se_x$ | | | | | | | |
|---|---|---|---|---|---|---|---|---|
| **Se content *x*** | $x$ = 0 | $x$ = 0.1 | $x$ = 0.2 | $x$ = 0.3 | $x$ = 0.5 | $x$ = 0.8 | $x$ = 1.5 | $x$ = 2.0 |
| **$E_g$ (eV)** | 0.72* | 0.69 | 0.72 | 0.73 | 0.63 | 0.64 | 0.63 | 0.63 |

* Data from the literature [41]

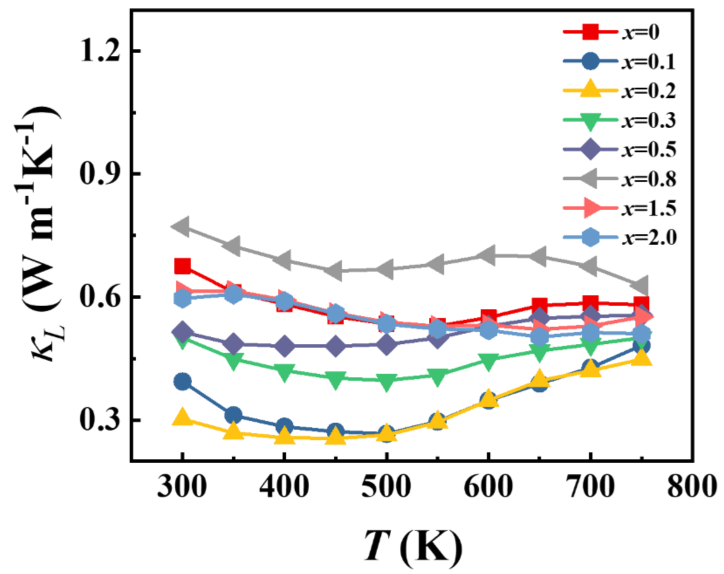


**Figure S4** Temperature dependence of lattice thermal conductivity $\kappa_L$ for $GeSb_4Te_{7-x}Se_x$ samples.

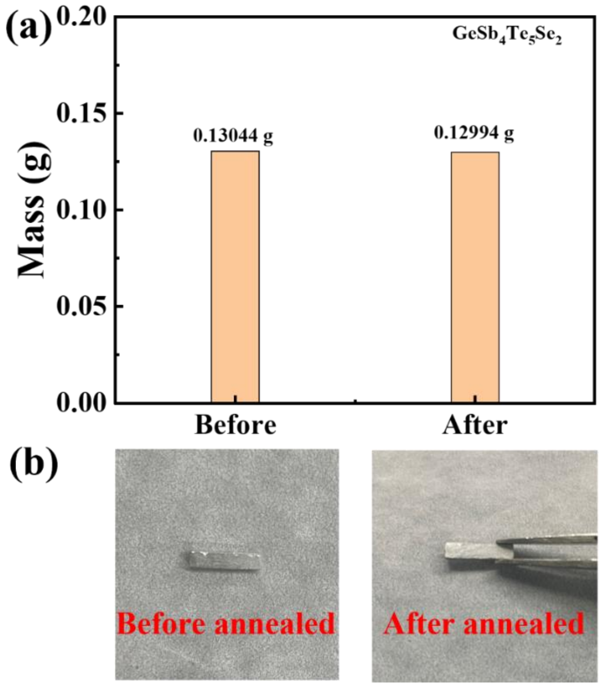


**Figure S5** (a) Mass change and (b) the surface of $GeSb_4Te_5Se_2$ samples before and after annealing at 750 K.
